# Associations of plasma homocysteine levels with peripheral systolic blood pressure and noninvasive central systolic blood pressure in a community-based Chinese population

**DOI:** 10.1038/s41598-017-06611-3

**Published:** 2017-07-24

**Authors:** Mohetaboer Momin, Fangfang Fan, Jianping Li, Xianhui Qin, Jia Jia, Litong Qi, Yan Zhang, Yong Huo

**Affiliations:** 1 Department of Cardiology, Peking University First Hospital, Beijing, China; 2 Renal Division, Nanfang Hospital, Southern Medical University, National Clinical Research Center for Kidney Disease, State Key Laboratory for Organ Failure Research, Guangzhou, China

## Abstract

Previous studies indicated that homocysteine (Hcy) is associated with higher peripheral systolic blood pressure (pSBP). There have been few data on the relationship between Hcy and central SBP (cSBP). A total of 4,364 Chinese subjects from the Shijingshan community in Beijing were included. cSBP and pSBP were measured with an Omron HEM-9000AI device. Subjects were 57.20 ± 8.9 years old, 37.9% were male. The median of Hcy was 11.96 μmol/L. The mean of cSBP and pSBP was 129.94 ± 18.03 mmHg and 133.25 ± 18.58 mmHg. lnHcy was associated with cSBP (adjusted β = 2.17, SE = 0.80, P = 0.007) and pSBP (adjusted β = 2.42, SE = 0.75, P = 0.001). With increasing Hcy, there were enhanced correlations of Hcy with pSBP and cSBP (p for trend between quartiles <0.01). Using Q1 for reference, the Q4 was associated with cSBP (adjusted β = 1.77, SE = 0.89, P = 0.047) and pSBP (adjusted β = 2.15, SE = 0.84, P = 0.011). The correlations were more significant in non-obese subjects than in obese subjects (cSBP: β = 4.30 vs 0.46, pSBP: β = 5.04 vs 1.18, P for interaction <0.001). Our study showed that Hcy was associated with higher cSBP and pSBP, especially in non-obese subjects.

## Introduction

Hyperhomocysteinemia (HHcy) has emerged as an independent risk factor for cardiovascular disease (CVD)^1^. However, whether homocysteine (Hcy) is a risk factor for hypertension still remains controversial. Several findings in conventional observational analyses supported a positive association between Hcy concentration and blood pressure (BP)^2–4^ as well as higher Hcy in hypertensive patients compared to normotensive patients in case-control studies^5–7^, but subsequent prospective studies yielded considerably weaker associations^8–10^. In contrast, observations that homocysteine-lowering therapies with folic acid-based treatments were associated with decreases in BP raise the possibility that the link between Hcy and BP is causal^11–14^.

Systolic pressure varies throughout the arterial tree; in most circumstances, aortic (central) systolic pressure (cSBP) is lower than the corresponding brachial values. However, some studies showed that more than 70% of individuals with high-normal BP had aortic systolic pressures that were similar to those of individuals with stage 1 hypertension^15^. Vital organs are exposed to the central rather than the peripheral BP although this difference is highly variable between individuals^16^. Emerging evidence now suggests that central pressure is better correlated with end-organ damage and cardiovascular events than peripheral systolic blood pressure (pSBP)^17^. Moreover, anti-hypertensive drugs can exert differential effects on brachial and central pressure^18^. Therefore, cSBP has different physiology and may offer improvements in CVD risk assessment compared to pSBP. Currently, cSBP can be assessed noninvasively through the use of several devices^19^. cSBP is associated with age, sex, brachial BP, heart rate, pulse wave velocity, and many other risk factors, such as body mass index (BMI), lipids and diabetes^20^. However there are few data about the relationship between Hcy and central BP.

The present study aims to elucidate the association of plasma Hcy with cSBP and pSBP in a Chinese community-based population.

## Methods

### Subjects

Participants were from the Gucheng and Pingguoyuan communities of the Shijingshan district in Beijing, China, and participated in an atherosclerosis cohort survey performed from December 2011 to April 2012. The methods and primary results of this survey have been reported elsewhere^21–23^. After excluding those with missing covariates, a total of 4,364 eligible participants aged ≥40 years old were included in this analysis. This study was approved by the ethics committee of Peking University and Peking University First Hospital, and each participant provided written informed consents before enrollment. We adhered to the principles of the Declaration of Helsinki. The procedures followed were in accordance with institutional guidelines.

### Data collection

Baseline data were collected by trained research staff according to standard operating procedures. All participants were interviewed using a standardized questionnaire that was specifically designed for the present study, providing information including sociodemographic status, education, occupation, diet, lifestyle, health behavior, medical history and medication use. Anthropometric measurements were taken according to a standard operating procedure. Current smoking was defined as smoking one cigarette per day for at least half a year. Current drinking was defined as drinking once per week for at least half a year. Diabetes was defined from self-reported history or index abnormality(fasting blood glucose (FBG) ≥ 7 mmol/L or oral glucose tolerance test (OGTT) ≥ 11.1 mmol/L); hypertension was defined from self-reported history or SBP ≥ 140 mmHg or DBP ≥ 90 mmHg. Dyslipidemia was defined from self-reported history or abnormal lipid profiles. CVD was defined as any self-reported history of coronary heart disease, myocardial infarction, stroke, or transient ischemic attack. BMI was calculated as weight (kg)/height^2^ (m^2^).

### Brachial blood pressure and central systolic blood pressure

Radial artery pressure waveforms and brachial BP were recorded simultaneously using a fully automated device (HEM-9000AI, Omron Healthcare, Kyoto, Japan) to calculate late systolic pressure in the radial artery (SBP2) and estimate central systolic BP. Brachial BP was measured with an oscillometric manometer and the radial pulse waveforms were recorded noninvasively using an applanation tonometer. Inflection points or peaks corresponding to early and late SBP were obtained from multidimensional derivatives of the original pulse waveforms. Then, the maximal SBP and DBP in the radial artery were calibrated with the brachial SBP and DBP. Finally, an estimate of cSBP was calculated by the pressure at the late systolic shoulder of the radial pressure waveform using linear regression with SBP2 as a major independent variable^24^.

### Blood sample collection and laboratory methods

A venous blood sample was obtained from the forearm of each participant after an overnight fast of at least 12 hours. Serum or plasma samples were separated within 30 minutes of collection and were stored at −80 °C. Plasma Hcy was measured using an autobiochemical analyzer (Beckman Coulter AU480) with the enzymatic method. This method mainly uses the S-adenosylhomocysteine (SAH) hydrolase reaction principle, in which SAH is hydrolyzed by hydrolytic enzymes into adenosine and Hcy, adenosine is immediately hydrolyzed into ammonia and hypoxanthine, nicotinamide adenine dinucleotide (NADH) is converted to NAD with ammonia and glutamic dehydrogenase, and the concentration of Hcy in the sample is proportional to the NADH transformation rate. Folate was measured using an automated chemiluminescence immunoassay analyzer (MAGLUMI4000) with the electrochemiluminescence method. Hcy and folate were all tested at the core laboratory of the National Clinical Research Center for Kidney Disease, at the Nanfang Hospital in Guangzhou, China. FBG and the standard 75-g OGTT as well as the lipid profiles and serum creatinine (Scr) at baseline were measured on the Roche C8000 Automatic Analyzer in the laboratory of the Chinese PLA General Hospital.

### Statistical analysis

Categorical variables were expressed as numbers and percentages. Continuous variables were described using means with standard deviations for data with normal distribution, and medians for non-normally distributed data. Univariate comparison were made between groups using ANOVA test for continuous variables and the χ^2^ test for categorical variables. A generalized additive model (GAM) with a spline smoothing function was applied to examine the relationship between cSBP, pSBP and Hcy, and a piecewise linear regression analysis was conducted to fit the smoothing curve, with adjustments for potential confounders, including age, sex, BMI, Scr, current smoking, current drinking, diabetes, dyslipidemia, CVD and antihypertension drug use. Univariate and multivariate analysis were performed to assess the associations between cSBP, pSBP and Hcy. The multivariable regression model was adjusted for other variables as well, including age, sex, BMI, Scr, current smoking, current drinking, diabetes, dyslipidemia, CVD and antihypertension drug use. Subgroup analyses examined the relationships of cSBP, pSBP and Hcy stratified by covariates, including sex, age, BMI, Scr, smoking, drinking, hypertension, diabetes mellitus, dyslipidemia, CVD, antihypertensive medication and folate. Tests for interactions in the linear regression model were used to compare β between the analyzed subgroups. Analyses were performed using Empower (R) (www.empowerstats.com, X&Y solutions, Inc. Boston MA) and R (http://www.R-project.org). A P-value < 0.05 was considered statistically significant.

### Declarations

Ethics approval, accordance and informed consent to participate:The proposal was approved by the ethics committee of Peking University and Peking University First Hospital, and all subjects signed informed consent before enrollment. We adhered to the principles of the Declaration of Helsinki. The procedures followed were in accordance with institutional guidelines.

## Results

The baseline characteristics of included participants stratified by Hcy quartiles are shown in Table 1. Subjects were 57.20 ± 8.91 years old, and 37.9% were male. The median value of Hcy was 11.96 (IQR: 10.03–14.92) μmol/L, and folate was 6.18 (IQR: 5.00–8.19) ng/ml. Of the subjects, 50.1% had hypertension, of whom 32.5% received antihypertensive medications. The mean value of cSBP and pSBP was 129.94 ± 18.03 mmHg and 133.25 ± 18.58 mmHg, respectively. To achieve an even distribution in each group, the subjects were divided into subgroups using Hcy quartiles: Q1: 8.87 (≤10.02) μmol/L; Q2: 11.02 (10.03–11.96) μmol/L; Q3: 13.16 (11.97–14.91) μmol/L; and Q4: 18.48 (≥14.92) μmol/L. Higher Hcy levels were significantly associated with female gender, older age, higher BMI, Scr, SBP, DBP and cSBP as well as a higher proportion of smokers, drinkers, subjects with hypertension, CVD and antihypertensive treatment usage. However, with increasing Hcy, folate levels have a downward trend (P < 0.001). No differences were observed for dyslipidemia or diabetes between groups. These data are presented in Table 1.

**Table 1:** Baseline Characteristics stratified by Hcy quartiles.

Hcy quartiles(μmol/L) Median	Total 11.96	Q1 8.87(≤10.02)	Q2 11.02(10.03–11.96)	Q3 13.16(11.97–14.91)	Q4 18.48 (≥14.92)	P-value
N	4364	1085	1097	1089	1093	
Age (year-old), mean ± SD	57.20 ± 8.91	53.76 ± 7.51	56.87 ± 8.08	58.90 ± 9.06	59.25 ± 9.76	<0.001
BMI (Kg/m^2^), mean ± SD	26.06 ± 3.37	25.88 ± 3.54	26.01 ± 3.31	26.08 ± 3.34	26.25 ± 3.29	0.016
Scr (μmol/L), mean ± SD	66.89 ± 15.57	57.38 ± 9.06	63.37 ± 12.01	68.88 ± 13.32	77.88 ± 18.39	<0.001
Sex, N (%)						<0.001
*Male*	1652 (37.90%)	117 (10.80%)	302 (27.50%)	486 (44.60%)	747 (68.30%)	
*Female*	2712 (62.10%)	968 (89.20%)	795 (72.50%)	603 (55.40%)	346 (31.70%)	
Current smoking, N (%)	848 (19.40%)	70 (6.50%)	152 (13.90%)	232 (21.30%)	394 (36.00%)	<0.001
Current alcohol drinking, N (%)	1022 (23.40%)	119 (11.00%)	210 (19.10%)	266 (24.40%)	427 (39.10%)	<0.001
Hypertension, N (%)	2188 (50.10%)	450 (41.50%)	523 (47.70%)	580 (53.30%)	635 (58.10%)	<0.001
Diabetes, N (%)	1089 (25.00%)	254 (23.40%)	288 (26.30%)	294 (27.00%)	253 (23.10%)	0.082
Hyperlipidemia, N (%)	3120 (71.50%)	773 (71.20%)	782 (71.30%)	800 (73.50%)	765 (70.00%)	0.342
Self-reported CVD, N (%)	574 (13.20%)	109 (10.00%)	132 (12.00%)	166 (15.20%)	167 (15.30%)	<0.001
Antihypertension Drugs, N (%)	1408 (32.50%)	284 (26.30%)	348 (32.00%)	367 (33.90%)	409 (37.70%)	<0.001
pSBP (mmHg)	129.94 ± 18.03	126.54 ± 17.33	128.70 ± 17.15	131.04 ± 18.11	133.48 ± 18.74	<0.001
cSBP (mmHg)	133.25 ± 18.58	131.60 ± 17.94	132.22 ± 17.87	133.89 ± 18.96	135.30 ± 19.31	<0.001
Hcy (μmol/L), Median (IQR)	11.96(10.03–14.92)	8.87(8.12–9.55)	11.02(10.52–11.47)	13.16(12.52–13.94)	18.48 (16.39–25.01)	<0.001
folate (ng/ml), Median (IQR)	6.18 (5.00–8.19)	7.71 (6.04–10.15)	6.68 (5.49–8.73)	5.94 (5.04–7.60)	4.90 (4.22–5.91)	<0.001

Abbreviations: Hcy, homocysteine; Scr, serum creatinine; BMI, body mass index; cSBP, central systolic blood pressure; pSBP, peripheral systolic blood pressure; CVD, cardiovascular disease.

**Figure 1:**
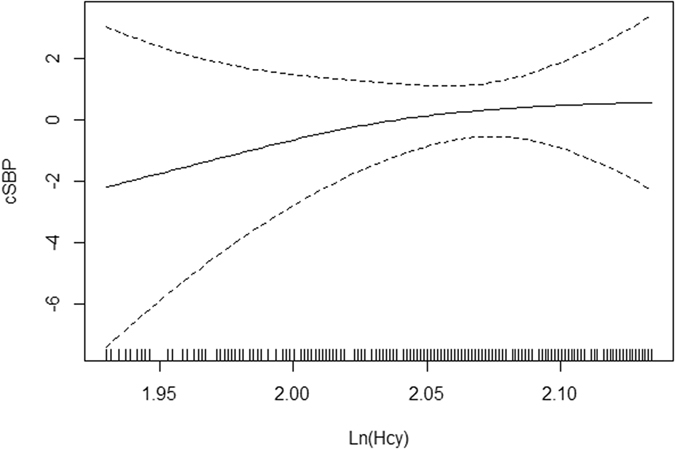
Smoothing curve of cSBP by lnHcy. Adjusted: age, sex, body mass index, smoking status, drinking status, serum creatinine, diabetes mellitus, dyslipidemia, cardiovascular disease, antihypertension drug use.

**Figure 2:**
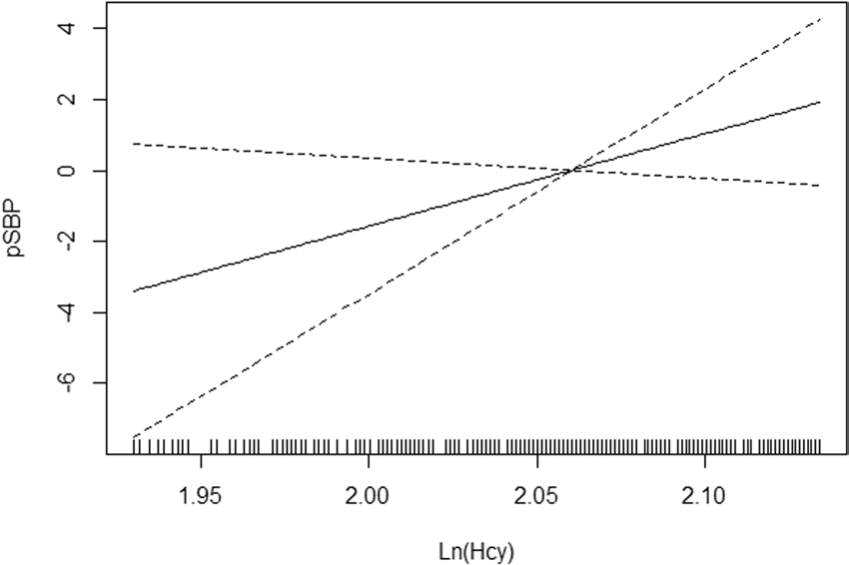
Smoothing curve of pSBP by lnHcy. Adjusted: age, sex, body mass index, smoking status, drinking status, serum creatinine, diabetes mellitus, dyslipidemia, cardiovascular disease, antihypertension drug use.

The smoothing curve showed that, after adjusting for confounders, including age, sex, BMI, smoking status, drinking status, Scr, DM, dyslipidemia, CVD and antihypertension drug use, there is a positive linear correlation between cSBP, pSBP and lnHcy, cSBP and pSBP were increasing linearly with lnHcy (Figs 1 and 2). Univariable and multivariable analyses were carried out to assess whether Hcy is independently associated with pSBP and cSBP after adjusting for likely confounders as mentioned above. For cSBP, lnHcy was positively associated with both cSBP and pSBP. A unit increase in lnHcy was associated with increases of 2.17 mmHg in cSBP (adjusted β = 2.17, SE = 0.80, P = 0.007) and increases of 2.42 mmHg in pSBP (adjusted β = 2.42, SE = 0.75, P = 0.001). With increasing Hcy, there were significantly enhanced correlations of Hcy with pSBP and cSBP (p for the trend between quartiles <0.01). Using Quartile 1(Q1) for reference, Quartile 4 (Q4) group was positively associated with both cSBP (adjusted β = 1.77, SE = 0.89, P = 0.047) and pSBP (adjusted β = 2.15, SE = 0.84, P = 0.011). These data are presented in Table 2. In addition to these confounders, we furtherly adjusted for folate, and the relationships of Hcy with pSBP and cSBP remained statistically significant. (for cSBP: adjusted β = 2.24, SE = 0.83, P = 0.007; for pSBP: adjusted β = 2.62, SE = 0.78, P < 0.001). Using Quartile 1(Q1) for reference, Quartile 4 (Q4) group was positively associated with both cSBP (adjusted β = 1.86, SE = 0.94, P = 0.047) and pSBP (adjusted β = 2.39, SE = 0.88, P = 0.007).

**Table 2:** Univariate and multivariate linear regression for effects of Hcy on cSBP and pSBP.

	cSBP				pSBP			
	Crude		Adjusted		Crude		Adjusted	
	β(SE)	P	β(SE)	P	β(SE)	P	β(SE)	P
LnHcy	3.02 (0.72)	<0.001	2.17 (0.80)	0.007	5.72 (0.69)	<0.001	2.42 (0.75)	0.001
Hcy quartiles								
Q1	0	—	0	—	0	—	0	—
Q2	0.62 (0.79)	0.435	−0.77 (0.78)	0.319	2.16 (0.76)	0.005	−0.15 (0.73)	0.838
Q3	2.29 (0.79)	0.004	0.36 (0.81)	0.659	4.50 (0.77)	<0.001	0.77 (0.76)	0.317
Q4	3.70 (0.79)	<0.001	1.77 (0.89)	0.047	6.95 (0.76)	<0.001	2.15 (0.84)	0.011
P for trend	<0.001		0.025		<0.001		0.007	

Abbreviations: Hcy, homocysteine; cSBP, central systolic blood pressure; pSBP, peripheral systolic blood pressure. Adjusted: age, sex, body mass index, smoking status, drinking status, serum creatinine, diabetes mellitus, dyslipidemia, cardiovascular disease, antihypertension drug use.

The interaction test showed no significant interactions between Hcy levels and the covariates mentioned above when different SBP traits were used to determine the outcomes, except for BMI (Table 3). The relationships were more significant in non-obese subjects than in obese subjects (for cSBP: β = 4.30 vs 0.46, P for interaction = 0.006; for pSBP: β = 5.04 vs 1.18, P for interaction = 0.004).

**Table 3:** Stratified and interaction analysis for effects of Hcy on cSBP and pSBP.

Subgroups(N)	cSBP			pSBP		
	Crude	Adjust	P interaction	Crude	Adjust	P interaction
	β(SE) P	β(SE) P		β(SE) P	β(SE) P	
**Age**						
<60 years old(2926)	2.02 (0.83) 0.015	2.08 (0.9) 0.020	0.900	4.78 (0.79) < 0.001	2.08 (0.9) 0.020	0.599
≥60 years old(1438)	2.81 (1.4) 0.044	2.28 (1.42) 0.109		4.65 (1.33) 0.001	2.28 (1.42) 0.109	
**Gender**						
Male(1652)	1.76 (1.06) 0.097	2.47 (1.04) 0.018	0.642	1.79 (1.02) 0.080	2.73 (0.98) 0.005	0.624
Female(2712)	5.78 (1.19) < 0.001	1.76 (1.19) 0.138		6.56 (1.15) < 0.001	2.02 (1.12) 0.071	
**BMI**						
BMI<25(kg/m^2^)(1740)	5.15 (1.13) < 0.001	4.30 (1.15) < 0.001	0.006	7.66 (1.09) < 0.001	5.04(1.09) < 0.001	0.004
BMI ≥ 25(kg/m^2^)(1624)	1.20 (0.92) 0.193	0.46 (0.97) 0.638		3.97 (0.88) < 0.001	1.18 (0.92) 0.196	
**Scr**						
Scr<64umol/L(2179)	4.20 (1.37) 0.002	1.44 (1.33) 0.279	0.443	5.69 (1.32) < 0.001	2.12 (1.25) 0.091	0.724
Scr ≥ 64umol/L(2185)	2.59 (0.96) 0.007	2.68 (0.97) 0.006		3.48 (0.93) < 0.001	2.66 (0.91) 0.004	
**Smoking status**						
Non-smoker(3516)	4.38 (0.91) < 0.001	1.46 (0.98) 0.134	0.331	7.50 (0.88) < 0.001	2.37 (0.92) 0.010	0.479
Smoker(848)	2.57 (1.34) 0.055	3.00 (1.29) 0.020		3.47 (1.29) 0.007	3.43 (1.22) 0.005	
**Drinking status**						
Non-drinker(3342)	4.08 (0.9) < 0.001	2.29 (0.97) 0.018	0.815	6.89 (0.87) < 0.001	2.55 (0.91) 0.005	0.800
Drinker(1022)	1.28 (1.3) 0.326	1.93 (1.27) 0.129		2.25 (1.25) 0.072	2.19 (1.2) 0.068	
**Diabetes**						
No(3275)	3.50 (0.79) < 0.001	2.43 (0.86) 0.005	0.416	6.1 (0.76) < 0.001	2.56 (0.81) 0.002	0.651
Yes(1089)	1.92 (1.62) 0.235	1.02 (1.62) 0.529		5.32 (1.55) 0.001	1.82 (1.52) 0.232	
**Dyslipidemia**						
No(1244)	4.1 (1.27) 0.001	2.06 (1.29) 0.110	0.916	6.66 (1.22) < 0.001	1.80 (1.21) 0.138	0.512
Yes(3120)	2.61 (0.87) 0.003	2.22 (0.92) 0.017		5.39 (0.84) < 0.001	2.71 (0.87) 0.002	
**CVD**						
No(3790)	3.26 (0.77) < 0.001	2.49 (0.84) 0.003	0.209	5.83 (0.74) < 0.001	2.65 (0.79) 0.001	0.336
Yes(574)	0.57 (2.03) 0.779	−0.13 (1.99) 0.949		3.92 (1.95) 0.045	0.77 (1.88) 0.682	
**Hypertension**						
No(2176)	0.57 (0.87)0.513	1.25 (0.91) 0.171	0.612	2.78 (0.79) 0.001	1.17 (0.82) 0.154	0.259
Yes(2188)	0.74 (0.87) 0.394	1.86 (0.93) 0.045		3.52 (0.79) < 0.001	2.40 (0.84) 0.004	
**Hypertension medication**						
No(2129)	2.49 (0.85) 0.003	2.24 (1.31) 0.086	0.942	5.01 (0.81) < 0.001	2.89 (1.23) 0.019	0.629
Yes(1408)	2.12 (1.26) 0.093	2.13 (0.92) 0.021		5.02 (1.2) < 0.001	2.21 (0.87) 0.011	

Adjusted: age, sex, body mass index, smoking status, drinking status, serum creatinine, diabetes mellitus, dyslipidemia, cardiovascular disease, antihypertension drug use. Abbreviations: Hcy, homocysteine; Scr, serum creatinine; BMI, body mass index; cSBP, central systolic blood pressure; pSBP, peripheral systolic blood pressure; DM, diabetes mellitus.

## Discussion

The major findings of our study were that Hcy levels were independently associated with both pSBP and cSBP, especially in non-obese subjects.

The relationship between HHcy and hypertension has been proposed by multiple researchers, most of whom only used brachial BP as the BP parameter. The results of the present study are consistent with some of the results from prior studies. Cross sectional data from the Third National Health and Nutrition Examination Survey showed that one standard deviation (5 μmol/l) increase in Hcy was associated with increases of 0.5 and 0.7 mmHg in diastolic and systolic blood pressure, respectively, after adjusting for cardiovascular risk factors^4^. The Hordaland study examined a very large sample (16, 176 individuals) and reported a weak association of plasma Hcy with SBP and DBP that was confined to younger individuals^2^. Data from a total of 3,524 schoolchildren including children and adolescents in a study of cardiovascular health showed that Hcy was independently associated with SBP^25^. Regina reported that SBP was correlated with Hcy levels and inversely correlated with plasma folates in juvenile essential hypertension patients^26^. Similar findings concerning the relationship between plasma Hcy and BP were provided by the SHEP study, which showed a direct correlation between Hcy and SBP in the elderly population^27^. A cross sectional study including 7,130 Chinese participants showed that HHcy was independently associated with the risk of hypertension in males (OR = 1.501, 95%CI: 1.012–2.227, P = 0.001)^3^. The relationship between SBP and Hcy was also found in hypertensive patients from Chinese rural areas^28^, smokers^29^, diabetic patients^30^, hemodialysis patients^31^, patients with stroke^32^, and several other small sample studies^5–7, 33^. On the other hand, numerous studies have yielded conflicting results. The Framingham Heart Study investigated for the first time in a community–based setting the relationships between plasma Hcy levels and hypertension. However, in age- and sex-adjusted analyses, the association was not statistically significant^34, 35^. In addition, no associations between SBP and DBP with Hcy concentrations were found in the Iranian population^36^, in Chinese subjects without antihypertensive medication use^37^, in young African American women^38^ or in the Brazilian population^39^. There were few prospective studies that illustrated the causal association between Hcy and BP. In Framingham Heart Study, no major relationship between baseline Hcy levels and hypertension incidence or longitudinal BP progression was found^8^. Wang reported that Hcy is related to hypertension incidence, with the results approximating a U-shaped curve in the Chinese population^9^.

Most interestingly, we find that Hcy is independently associated with cSBP. The studies addressing the link between central arterial BP and Hcy were very limited. Xiao *et al*. reported that, in a cross-sectional study with a community-based sample of 1680 Chinese subjects, neither peripheral nor central BP differed according to Hcy levels in normotensive and hypertensive subjects^40^. The BROOF study demonstrated that lnHcy was strongly associated with PWV, but no significant association was observed for Aix and aortic pulse pressure^41^. To the best of our knowledge, this is the first study to report the positive relationship between the Hcy level and cSBP. The results may have some instructive significance. First, mechanisms that could explain the relationship between Hcy and BP include homocysteine-induced arteriolar constriction, renal dysfunction, increased sodium reabsorption, and increased arterial stiffness^42, 43^. Compliance of central artery is one of the most important factors that influence cSBP. Thus, the results of the study indicate that the arterial stiffness might be an important issue linking HHcy and hypertension. In addition, it has been established that HHcy is a risk factor for CVD, and the association between Hcy and cSBP may contribute to the elevated CVD risks that HHcy induced.

We also found that Hcy levels were more associated with both cSBP and pSBP in the non-obese subgroup. There were very few prior studies that could explain these results, so we raise some hypotheses. First, obesity is considered a risk factor for hypertension and other CVD related factors, therefore, in higher BMI groups, the association between Hcy and SBP might be negated by other factors. Second, studies have reported that Hcy is associated with insulin resistance^44, 45^, which hypertension is one of the features of this syndrome. And this may link the Hcy and hypertension in non-obese population. Third, The sympathetic nervous system is an important regulator of blood pressure, especially in non-obese subjects, but the effects of Hcy on its activity do not appear to have been studied. The interference of BMI to Hcy and hypertension needs more basic and independent sample researches to be further verified.

Compared to prior studies, the population of our study was from a Chinese urban community, which is not covered as much in previous studies. The sample size of our study is relatively larger. The median level of Hcy was 11.98 μmol/L, which was comparable to the other data^46^. The prevalence of hypertension is similar to that reported in prior research^3^. However, the proportion of diabetes is relatively higher. Moreover, as previous observation studies have shown that the relationship between BP and Hcy attenuated after adjustments, it is possible that plasma Hcy is a marker for age, age-related renal dysfunction and hypertensive drugs with Hcy-elevating effects^47^. Thus, many factors that might have contributed to hypertension and HHcy were taken into count in the present study, and after adjustments and subgroups analyses, the association remained statistically significant. Furthermore, we assessed the association using both pSBP and cSBP, which provided more solid evidence in support of the findings from previous studies.

The present study has several limitations. First, it was a cross-sectional study, and thus, predictions about the incidence of hypertension due to HHcy in the general population cannot be extrapolated from these data. Longitudinal studies are required for the further investigation of these findings. Second, the data are not necessarily representative of populations in other locations within China, but many studies conducted in different regions reported data that were consistent with ours. Third, pSBP and cSBP values were based on a single assessment, which may introduce variation, but the large sample of the study can also attenuate the variation. In addition, antihypertensive drugs, and particularly beta-blockers, exert differential effects on central blood pressure, but we weren’t able to detail the effects of antihypertensive drugs. Fourth, other Hcy related factors, including lifestyle factors such as coffee consumption and physical activity, genetics data, vitamin B intake except folate, that were not assessed in detail due to lack of such data.

## Conclusion

In conclusion, we found that plasma Hcy levels are independently associated with pSBP and cSBP especially in non-obese subjects, which provide potential evidence that Hcy may play an important role in regulating blood pressure and hypertension. Large prospective studies and independent replications are required to elucidate these issues.
